# Parent-reported Areas of Greatest Challenge for their ADHD and/or Autistic Children

**DOI:** 10.1007/s41252-024-00417-x

**Published:** 2024-09-11

**Authors:** Willow J. Sainsbury, Andrew J. O. Whitehouse, Kelly D. Carrasco, Hannah Waddington

**Affiliations:** 1 Faculty of Education and Social Work, University of Auckland, Auckland, New Zealand; 2 Telethon Kids Institute, Perth, Australia; 3 Department of Psychology, California State University, Fresno, USA; 4 Faculty of Education, Victoria University of Wellington, Wellington, New Zealand

**Keywords:** Autism, ADHD, Challenges, Family-life, Co-occurring, AuDHD, Factors influencing quality of life, Stress

## Abstract

**Objectives:**

This study aimed to understand how parents describe the most challenging behaviors exhibited by their children diagnosed with autism and/or ADHD, how those behaviours impact their family, and whether challenges are directly related to the core characteristics of these conditions.

**Methods:**

A total of 258 New Zealand parents of children diagnosed with autism (*n* = 98), ADHD (*n* = 85), or both conditions (*n* = 75) answered an open-ended question about what child behaviour(s) provide the most challenge for their child/family’s life. Responses were coded into 13 domains.

**Results:**

The areas of greatest challenge fitted with the core characteristics of the respective diagnoses, but the co-occurring group favoured greater challenges in the autism domain. Emotional dysregulation challenges were substantial across all three groups. A significant predictor of parents reporting challenges in the autism and internalising domain was a higher age.

**Conclusions:**

These results have the potential to inform more targeted supports for children and families by considering what is important to parents.

**Supplementary Information:**

The online version contains supplementary material available at 10.1007/s41252-024-00417-x.

Autism and attention deficit hyperactivity disorder (ADHD) are neurodevelopmental conditions that occur early in childhood and have a high male-to-female diagnosis ratio (Antshel et al., 2016). Autism is characterised by focused interests and strict adherence to routines and differences in social communication and interaction, while ADHD involves attention difficulties, impulsivity and hyperactivity (Association Psychiatric Association A, 2013). The conditions have high rates of co-occurrence with 40–70% of autistic children also having ADHD (Antshel et al., 2016).

There is a growing body of research that has highlighted the importance of a strength-based approach to parenting an autistic child (Clark & Adams, 2020; Ekas et al., 2015; Lee et al., 2020). However, there are also difficulties in parenting a neurodivergent child, including social pressures, and challenges in accessing resources to support the child and family (Sainsbury et al., 2023). Indices of these strengths and difficulties can be gauged by measuring parent quality of life (hereafter QoL) or stress which is known to correlate with lower QoL (Iadarola et al., 2019; Leitch et al., 2019; Vasilopoulou & Nisbet, 2016). In a study measuring health-related QoL across physical, psychological, social and environmental domains of parents of autistic children, “severity of autism” and “behaviour problems” were moderately associated with lower QoL, but only parent stress was significant across all domains explaining 42–47% of the variance in the model (Tung et al., 2014). However, understanding how parents subjectively assess the causation of their stress or how behaviour problems/severity are conceived by parents, in relation to diagnostic characteristics across multiple neurodevelopmental conditions, might further add to this understanding of how to target support for improved QoL and reduced parenting stress.

Three parental-stress studies specifically compared the diagnostic groups; autism, ADHD and autism + ADHD (May & Williams, 2022; Miranda et al., 2015; van Steijn et al., 2014) and found significantly increased parenting stress compared with controls, but no significant differences across the three groups in terms of parenting stress. However, May and Williams (2022) did find that in the co-occurring diagnosis group, maternal stress levels peaked earlier. One contributing factor to parenting stress outlined in the literature on parenting autistic and ADHD children is described as, “challenging behaviour” or “emotional and behavioural problems” experienced by the child (Leitch et al., 2019; Mofokeng & van der Wath, 2017; Ooi et al., 2016). This behaviour is rarely described in detail, but when described, these challenges/problems often do not relate to the core diagnostic characteristics of either autism or ADHD (Leitch et al., 2019; Mofokeng & van der Wath, 2017; Ooi et al., 2016). Two qualitative autism studies asked specifically, “As a parent of a child with autism, what makes things difficult or challenging for you?” (Ludlow et al., 2012) and “How has your child in the autism spectrum affected your life and your family's life? (Myers et al., 2009 p. 670). While these studies identified “challenging behaviour” related to parenting an autistic child, none identified the area or areas of greatest challenge, compared this across the different diagnosis groups, or looked for a relationship of the description to the expected diagnostic characteristics.

The current study asked parents of children diagnosed with autism and/or ADHD what behaviours of their child provide the most challenge for their family/child’s life in order to understand how parents subjectively evaluated what lowered their QoL. The study’s first hypothesis was that the greatest challenge might not represent the diagnostic characteristics of either autism or ADHD for any of the groups (Leitch et al., 2019; Mofokeng & van der Wath, 2017; Ooi et al., 2016). If the core characteristics of the diagnosis are not relevant to the greatest challenge, this might lead to different targeted supports for the family. The second hypothesis was that the co-occurring group (autism + ADHD) might resemble an ADHD diagnostic-group pattern of most challenging behaviour due to the phenomena of ADHD often being diagnosed before autism, when present in children (Sainsbury et al., 2022). ADHD is often diagnosed before autism because the characteristics presented to diagnosticians by the parent emphasise more of the ADHD pattern, therefore, it follows that parents might present the most challenge for their family life upon seeking a diagnosis (Sainsbury et al., 2022). Understanding the areas of greatest challenge experienced by parents would allow practitioners to prioritise and tailor support and also elucidate differences in the presentation of co-occurring autism + ADHD.

## Methods

### Participants

Legal guardians, caregivers and parents (hereafter parents) of children diagnosed with autism, ADHD and both conditions (autism + ADHD) were invited to participate in an online survey which was voluntary and anonymous. Participants were recruited using convenience sampling, including emails sent from Autism or ADHD New Zealand and online specialised social media groups. To be eligible, participants needed to be (a) living in New Zealand and (b) the parent of a child aged 18 years or younger who had a clinical diagnosis of autism, ADHD, or autism + ADHD. Ethical approval was granted by the Human Ethics Committee at Victoria University of Wellington, New Zealand [Approval number 28993]. Informed consent was obtained from all participants at the beginning of the questionnaire and participation was voluntary and anonymous.

### Procedures and Materials

The survey was developed by the first author based on The Australian Autism Biobank’s Family History Questionnaire (Alvares et al., 2018). The survey was revised after consultation with a research and advocacy advisor at Autism New Zealand and other authors of the study. The survey was hosted on Qualtrics from the 15th of March until the 1st of June, 2021. Parents took approximately fifteen minutes to complete the survey. The survey comprised 30 questions, of which only the nine demographic questions and the specific question on challenges were relevant to the current study. The demographic characteristic questions included parent ethnicity, relationship to the child, highest education level, and total household income. Child demographic characteristics included gender, age, the presence and birth position of siblings, and age of diagnosis. There was also a question asking parents if their child had any other diagnoses with options listed and a free-text “other” box. The question pertaining to areas of greatest challenge asked “What behaviours of your child provide the most challenge for your family/child's life?”.

### Data Coding

Parent free-text responses regarding areas of greatest concern were inductively coded into categories by W.J.S. (Smith & Firth, 2011). New categories were created until the saturation of parents’ input was achieved (Smith & Firth, 2011). W.J.S. and H.W. then checked for overlap in categories (hereafter sub-domains). A framework was then developed which created domains based on diagnostic criteria of autism and ADHD, and further psychological concepts of behaviour (Smith & Firth, 2011). The final domains were autism characteristics, ADHD characteristics, communication differences, externalising behaviour, internalising behaviour, emotional dysregulation, sensory issues, sleep difficulties, toileting issues, elopement, cognitive and developmental difficulties, societal issues and others. Eight of these domains had at least two or more subdomains. Each parent response was coded for all relevant domains and subdomains. A score of 1 in each domain/subdomain indicated that the challenge was present in the response, while a score of 0 indicated that it was not. It was possible for a parent to challenge several subdomains within each domain. W.J.S. independently coded all parents’ responses and H.W. independently coded 20% of these responses, disagreements were resolved by consensus. The domain-level agreement ranged from 93.33 to 100%. The subdomain level agreement ranged from 93.33 to 100%.

### Data Management and Analysis

Descriptive statistics were used for the demographic data and for the number of participants reporting each area of greatest challenge. Household income data collapsed from 13 $NZ 10,000 increments to four $NZ 50,000 increments. None of the “Other” ethnicities reached a threshold for analysis and were thus grouped together. Each participant’s ethnicity was categorised as “Māori” (the indigenous people of New Zealand) if one of their stated ethnicities was Māori.

All inferential data analysis was performed using IBM SPSS Statistics (version 28). Binary logistic regression analyses were used to examine the association between demographic characteristics and domains of greatest challenge. For domains in which ≥ 50 participants reported that the challenge was present (autism characteristics; ADHD characteristics, externalising behaviour, internalising behaviour, emotional dysregulation, sensory), predictors included child age, gender, ethnicity and diagnosis (autism, ADHD, or autism + ADHD); and family income. A Hosmer–Lemeshow goodness-of-fit test was simultaneously performed and the Nagelkerke *R*^2^ value was reported. For each analysis, age was found to be linearly related to the logit of the dependent variable, assessed via the Box-Tidwell procedure. In line with existing guidance (Peduzzi et al. 1996), in domains in which 10–49 participants reported that the challenge was present (communication, sleep issues, elopement), child diagnosis was retained as the sole predictor. The toileting, cognitive and societal issues domains were not analysed because fewer than 10 participants reported these as areas of greatest challenge.

## Results

### Sample Characteristics

The parents of 355 children participated in the survey. Of these, 97 parents were excluded due to not reporting a diagnosis for their child or not completing free-text questions on challenges, leaving a total of 258 participants. The participants were then divided into parents of children with either autism (*n* = 98); ADHD (*n* = 85); autism + ADHD (*n* = 75).

Table 1 provides the child and parent demographic information. Overall, children were predominantly male, with a mean age of 110 months (9.17 years). The majority of parents were New Zealand European mothers, who had attended university, with a household income of between $NZ 50,000–99,000. The average income in New Zealand per person is $NZ 68,000 (OECD, 2022).

**Table 1:** Child and parent demographic characteristics (*n* = 258)

Demographic characteristic	Total *n* (%)/mean (months) (SD)	Autism only *n* (%)/median (months) (SD)	ADHD only *n* (%)/median (months) (SD)	Autism + ADHD *n* (%)/median (months) (SD)	Missing data (*n*)
Participants per group Child gender	*n* = 258	*n* = 98	*n* = 85	*n* = 75	
Male	195 (76.2%)	71 (73.2%)	63 (75.0%)	61 (81.3%)	
Female	56 (21.9%)	23 (23.7%)	20 (23.8%)	13 (17.3%)	
Non-binary	5 (2.0%)	3 (3.1%)	1 (1.3%)	1 (1.3%)	
Prefer not to say	2 (0.7%)	1 (1%)	1 (1.3%)	0 (0%)	
Child chronological age (months)	110.00 (2.7)	86.50 (4.9)	108.00 (3.9)	126.0 (4.3)	
Child age of autism diagnosis (months)		48.00 (4.1)		84.00 (4.5)	
Child age of ADHD diagnosis (months)			84.00 (3.1)	81.00 (3.1)	
Parent ethnicity					1
NZ European (Pākehā)	181 (70.2%)	64 (65.3%)	65 (76.5%)	52 (69.3%)	
Māori	58 (22.5%)	25 (25.5%)	12 (14.1%)	21 (28.0%)	
Other	19 (7.4%)	9 (9.2%)	8 (9.4%)	2 (2.7%)	
Parent relationship to child					
Biological mother	246 (95.3%)	95 (96.9%)	80 (94.1%)	71 (94.7%)	
Biological father	5 (1.9%)	1 (1%)	4 (4.7%)	0 (0%)	
Legal guardian/caregiver	7 (2.7%)	2 (2%)	1 (1.2%)	4 (5.3%)	
Parent highest education					
Secondary school	30 (11.7%)	11 (11.2%)	11 (13.1%)	8 (10.7%)	
Trade/diploma	83 (32.3%)	34 (34.7%)	24 (28.6%)	25 (33.3%)	
University	105 (40.9%)	39 (39.8%)	37 (44.0%)	29 (38.7%)	
Postgraduate	19 (7.4%)	7 (7.1%)	5 (6.0%)	7 (9.3%)	
Other or prefer not to say	20 (7.8%)	7 (7.1%)	7 (8.3%)	6 (8.0%)	
Household income $NZ					
< $50,000	53 (20.5%)	18 (18.4%)	9 (10.6%)	26 (34.7%)	
$50,000–$99,999	96 (37.2%)	40 (40.8%)	36 (42.4%)	20 (26.7%)	
$100,00–149,999	48 (18.6%)	19 (19.4%)	15 (17.6%)	14 (18.7%)	
$150,000 or more	45 (17.4%)	16 (16.3%)	18 (21.2%)	11 (14.7%)	
Prefer not to say	16 (6.2%)	5 (5.1%)	7 (8.2%)	4 (5.3%)	
Co-occurring diagnoses					
Intellectual disability/global Developmental delay	37 (14.3%)	13 (13.3%)	9 (10.6%)	15 (20.0%)	
Learning difficulty	52 (20.2%)	9 (9.2%)	23 (27.1%)	20 (26.7%)	
Oppositional defiance disorder	40 (15.5%%)	3 (3.1%)	26 (30.6%)	11 (14.7%)	
Sensory processing disorder	84 (32.6%)	42 (42.9%)	15 (17.6%)	27 (36.0%)	

*Note*: Parent education and parent ethnicity relate to the parent who completed the survey. Pākehā is an Indigenous term used for New Zealanders of European descent

Other includes: Pacific People, Asian, European, South African, American, South American, Middle Eastern, African

There were similar rates of intellectual disability (ID) across the diagnostic groups with a total of 14.3% reporting an intellectual disability. The ADHD group reported the highest in oppositional defiance disorder and learning difficulties, with autism the highest in the sensory processing disorder category for co-occurring diagnoses.

### Areas of Greatest Challenge

The percentage of parents reporting a challenge in each domain and subdomain is reported in Table 2. Across all groups, the most frequently included greatest challenges were autism characteristics, emotional dysregulation and externalising behaviour. For the autism group, the most frequently included greatest challenges were autism characteristics, followed by emotional dysregulation, and then internalising behaviour. For the ADHD group, the most frequently included challenges were ADHD characteristics followed by externalising behaviour and emotional dysregulation. For the autism + ADHD group, the most frequently included challenges were autism characteristics, externalising behaviour, and emotional dysregulation.

**Table 2:** Percentage of parents reporting areas of greatest challenge in each domain and subdomain across diagnostic groups (*n* = 258)

	All diagnostic groups *n* (% of total)	Autism only *n* (% of group)	ADHD only *n* (% of group)	Autism + ADHD *n* (% of group)
Participants per group	*n* = 258	*n* = 98	*n* = 85	*n* = 75
Domain/subdomain				
ADHD characteristics	**84 (32.6%)**	**11 (11.2%)**	**52 (61.2%)**^**1**^	**21 (28.0%)**
Hyperactivity	24 (9.3%)	2 (2.0%)	15 (17.6%)	7 (9.3%)
Attention	36 (13.9%)	4 (4.1%)	23 (27.1%)	9 (12.0%)
Impulsivity	46 (17.8%)	6 (6.1%)	28 (32.9%)	12 (16.0%)
Autism characteristics	**99 (38.2%)**	**43 (43.9%)**^**1**^	**20 (23.5%)**	**36 (48.0%)**^**1**^
Social differences	54 (20.8%)	16 (16.3%)	16 (18.8%)	22 (29.3%)
Fixed rigid behaviour	39 (15.1%)	21 (21.4%)	4 (4.7%)	14 (18.7%)
Difficulty with everyday routines	11 (4.2%)	6 (6.1%)	3 (3.5%)	2 (2.7%)
Difficulty with social communication	13 (5.0%)	9 (9.2%)	1 (1.2%)	3 (4.0%)
Communication	**16 (6.2%)**	**14 (14.3%)**	**1 (1.2%)**	**1 (1.3%)**
Externalising behaviour	**91 (35.1%)**	**25 (25.5%)**	**34 (40.0%)**^**2**^	**32 (42.7%)**^**2**^
Demand avoidance issues	31 (12%)	6.1% (6)	15 (17.6%)	10 (13.3%)
Confrontational	12 (4.6%)	1 (1.0%)	9 (10.6%)	2 (2.7%)
Destruction of property	5 (1.9%)	1 (1.0%)	4 (4.7%)	0.0%
Shouting and screaming	8 (3.1%)	2 (2.0%)	4 (4.7%)	2 (2.7%)
External control	6 (2.3%)	3 (3.1%)	1 (1.2%)	2 (2.7%)
Aggression towards others	53 (20.5)	16 (16.3%)	17 (20.0%)	20 (26.7%)
Internalising behaviour	**58 (22.4%)**	**34 (34.7%)**^**3**^	**8 (9.4%)**	**16 (21.3%)**
Moodiness	4 (1.5%)	3 (3.1%)	1 (1.2%)	0 (0.0%)
Self-harm	5 (1.9%)	3 (3.1%)	1 (1.2%)	1 (1.3%)
Suicidal thoughts	1 (0.4%)	1 (1.0%)	0 (0.0%)	0 (0.0%)
Avoidant social environments	19 (7.3%)	14 (14.3%)	2 (2.4%)	3 (4.0%)
Anxiety	30 (11.6%)	18 (18.4%)	2 (2.4%)	10 (13.3%)
Obsessive behaviour	8 (3.1%)	3 (3.1%)	2 (2.4%)	3 (4.0%)
Emotional dysregulation	**97 (37.5%)**	**35 (35.7)**^**2**^	**33 (38.8)**^**3**^	**29 (38.7)**^**3**^
Anger issues	14 (5.4%)	6 (6.1%)	6 (7.1%)	2 (2.7%)
Emotional dysregulation	40 (15.4%)	10 (10.2%)	23 (27.1%)	7 (9.3%)
Meltdowns/tantrums	53 (20.5%)	24 (24.5%)	8 (9.4%)	21 (28.0%)
Sensory	**54 (20.8%)**	**25 (25.5%)**	**11 (12.9%)**	**18 (24.0%)**
Sensory seeking-noise	12 (4.6%)	4 (4.1%)	6 (7.1%)	2 (2.7%)
Sensory food	18 (6.9%)	11 (11.2%)	3 (3.5%)	4 (5.3%)
Other sensory	31 (12.0%)	15 (15.3%)	3 (3.5%)	13 (17.3%)
Sleep	**23 (8.9%)**	**9 (9.2%)**	**7 (8.2%)**	**7 (9.3%)**
Toileting	**6 (2.3%)**	**4 (4.1%)**	**1 (1.2%)**	**1 (1.3%)**
Elopement	**19 (7.3%)**	**11 (11.2%)**	**4 (4.7%)**	**4 (5.3%)**
Safety concerns	9 (3.5%)	6 (6.1%)	3 (3.5%)	0 (0.0%)
Running away	11 (4.2%)	6 (6.1%)	1 (1.2%)	4 (5.3%)
Cognitive issues	**8 (3.1%)**	**2 (2.0%)**	**2 (2.4%)**	**4 (5.3%)**
Cognitive differences	7 (3.8%)	2 (2.0%)	2 (2.4%)	3 (4.0%)
Learning difficulty	2 (0.8%)	0 (0.0%)	0 (0.0%)	2 (2.7%)
Societal issues	**4 (1.5%)**	**0 (0.0%)**	**2 (2.4%)**	**2 (2.7%)**
Other (e.g., auditory processing, migraines)	**13 (5.0%)**	**7 (7.1%)**	**5 (5.9%)**	**1 (1.3%)**

*Note*: Items in bold are domains while indented and non-bolded items are subdomains

Superscript numbers rank 1–3 of the most common areas of challenge for each diagnostic group

### Predictors of Areas of Greatest Challenge

#### Multiple Demographic Characteristics

Binary logistic regressions were used to examine demographic predictors of areas of greatest challenge. The combined demographic characteristics were significant predictors of the presence or absence of concern in the autism domain (*p* < 0.001, see Supplementary Table S1.). Within the model, increasing child age, being male compared to female, being diagnosed with autism + ADHD compared to being diagnosed with ADHD only, and earning > 150 K compared to earning < 50 K were all associated with an increased likelihood of reporting autism characteristics as an area of greatest challenge.

In the ADHD domain, the overall regression was statistically significant (*p* < 0.001, see Supplementary Table S2). It was found that a child being diagnosed with autism + ADHD significantly predicted parents indicating the ADHD domain compared with the autism-diagnosed group (*p* = 0.002), but significantly less compared with the ADHD-diagnosed group (*p* =  < 0.0 × 01).

In the externalising domain, there were no cases of non-binary (gender) selected, so this variable did not fit the model and therefore, this group was excluded. The overall regression was not significant for this domain (*p* = 0.246, see Supplementary Table S3). However, it was found that a child being diagnosed with autism + ADHD significantly predicted parents indicating an externalising domain compared with the autism-diagnosed group (*p* = 0.024).

In the internalising domain, the overall regression was significant (*p* < 0.001; see Supplementary Table S4). As the age of the child increases the model significantly predicted that a parent would report challenges related to the internalising domain (*p* = 0.045). A child being diagnosed with autism significantly predicted parents indicating the internalising domain compared with the autism + ADHD diagnosed group (*p* = 0.001).

In the emotional dysregulation domain and the sensory issues domain, the overall regressions were not significant (respectively, *p* = 0.365, see Supplementary Table S5; *p* = 612, see Supplementary Table S6).

#### Diagnosis Only

Binary logistic regression analyses were conducted to examine whether the diagnosis was a significant predictor of challenges associated with communication, sleep issues and elopement (see Supplementary Tables S7–9). The only significant finding was for the communication domain. A child being diagnosed with autism significantly increased the likelihood of parents indicating communication as an area of greatest challenge compared with the autism + ADHD diagnosed group (*p* = 0.016).

## Discussion

The results of this study indicate that significant challenges for parents of children diagnosed with autism, ADHD and both diagnoses related to the characteristics of autism and/or ADHD, but the parents frequently reported finding emotional dysregulation and either internalising or externalising behaviour a significant challenge. There are also specific patterns which pertain to the diagnostic categories. Parents in the co-occurring group reported more challenges related to autism characteristics than ADHD. This was surprising given the theory that ADHD characteristics can mask the child's autism characteristics (Miodovnik et al., 2015; Sainsbury et al., 2022). Of particular note, was the subcategory of social differences, which was higher in the co-occurring group than the autism-only group. Subgroups in ADHD characteristics were also unexpected with attention and impulsivity more commonly reported than hyperactivity.

Challenges with externalising behaviour were significantly lower in the autistic-only diagnostic group compared to the ADHD and the co-occurring groups. Again, significant differences across the diagnostic subtypes were not expected due to previous research not finding significant differences (May & Williams, 2022; Miranda et al., 2015; van Steijn et al., 2014). This pattern of lower autism externalising behaviour compared to the other diagnostic groups was inverted for the internalising behaviour domain with the autistic group significantly higher than the ADHD and co-occurring diagnostic group. Although not reported in previous parent studies, these findings are similar to clinical assessments of these three diagnostic groups in terms of observed externalising and internalising behaviour (Craig et al., 2015). Therefore, it is interesting to note that parents in this study reported similar patterns to clinical observations when describing challenges for their child or family.

Specific support for families parenting an autistic + ADHD child is limited (Antshel et al., 2016) and one implication of this study is that tailored support might help parents to have more understanding of core characteristics and help parents to adapt their own approach or the environment to better cope with these diagnostic differences and increase quality of life (Vasilopoulou & Nisbet, 2016). The aim of support might also follow a neurodiversity approach of reframing these challenges as differences (Sonuga-Barke & Thapar, 2021). This study also indicates that greater support in areas that sit outside diagnostic criteria might be beneficial, such as supporting emotional regulation and reducing externalising and internalising behaviour issues (Antshel et al., 2016). The perceived challenges of the child that are most pertinent to the parent should also indicate a readiness for parents to obtain support in these particular areas.

The age might also be considered when tailoring support with more internalising and autistic domain behaviours occurring with older children. It was expected that more complex internalising behaviour might occur with age (Gutman & Codiroli McMaster, 2020); however, the autism behaviour being positively correlated with increasing age is surprising due to autistic traits apparent from an earlier age than ADHD (Hyman et al., 2020).

One unique aspect of the study is that the domain and subdomains of challenges were parent-generated. A systematic review of stress related to parenting an autistic child, reports that studies included problem behaviour and autism “severity” scales, but this research was not able to capture the perception of the parents as to what they perceived as the most pressing, or stressful, behavioural challenge of their child (Enea & Rusu, 2020). If conceived of in layers, the “problem behaviour” and “autism severity” contribute to parental stress which in turn reduces the QoL of the family. There is academic debate about how best to define and measure QoL (Barcaccia et al., 2013); however, it is important that contributing factors to QoL continue to be subjectively parent-defined. This enables more comprehensive descriptors to be added to subjective evaluations of how parents perceive their child’s behaviour and that support has meaning or specific relevance to what parents understand reduces their QoL. An intervention that is specifically relevant to a parental-perceived challenge might result in enhanced family participation, reduced stress and improved QoL.

One limitation of this study is that the participant cohort over-indexed participants from higher socio-economic groups and mothers. The majority of participants were university-educated mothers. Parents language was particularly noticeable whereby psychology terms were regularly used, such as “emotional dysregulation”, “executive function”, “sensory issues,” “survival-brain behaviour,” and “locus-of-control.” Parents also approached the question in multiple ways such as indicating the possible cause of the behaviour, or the outcome of the cause, as the greatest challenge. Finally, a limitation of this study is that it is focused on challenges and neglects the many positives that come with parenting a neurodivergent child. Future research might consider the greatest perceived challenges and strengths of parenting a child with autism, ADHD and/or autism + ADHD.

## Supplementary Information

Below is the link to the electronic supplementary material.

Supplementary file1 (DOCX 55 KB)

## Data Availability

The participants of this study did not give consent for their data to be shared publicly, and due to the open text response the data is not available. Most of the data supporting the findings of this study are available within the article and its supplementary materials. Coding book and further coding examples that support the findings of thisstudy are available on request from the corresponding author.
