# Supplementary material for: Parent-reported Areas of Greatest Challenge for their ADHD and/or Autistic Children
Source: Adv Neurodev Disord. 2024 Sep 11;9(2):330–7. doi: 10.1007/s41252-024-00417-x (PMC12222311; doi:10.1007/s41252-024-00417-x)
Supplement: Supplementary file 1 — Supplementary file1 (DOCX 55 KB) [file 41252_2024_417_MOESM1_ESM.docx]

Supplementary Table 1.

*Logistic regression of Autism domain across demographic and diagnostic categories*

|  | **Autism Characteristics** | | | | | | | |
| --- | --- | --- | --- | --- | --- | --- | --- | --- |
| Predictors | β | S.E | Wald | df | *p* | Exp (β) | *95% C.I for Exp* (β) | |
|  |  |  |  |  |  |  | *Lower* | Upper |
| Age | .007 | .003 | 4.434 | 1 | **.035** | 1.007 | 1.000 | 1.014 |
| Gender |  |  | 6.149 | 2 | **.046** |  |  |  |
| Male v. female | .789 | .361 | 4.767 | 1 | **.029** | 2.201 | 1.084 | 4.467 |
| Gender diverse v. female | -.776 | 1.219 | .405 | 1 | .524 | .460 | .042 | 5.019 |
| Ethnicity |  |  | 1.596 | 2 | .450 |  |  |  |
| Māori v. NZ European | -.448 | .356 | 1.581 | 1 | .209 | .639 | .318 | 1.285 |
| Other v. NZ European | -.154 | .536 | .082 | 1 | .774 | .857 | .300 | 2.453 |
| Level of Income |  |  | 10.197 | 4 | **.037** |  |  |  |
| 50 - 100 K v. < 50 K | .670 | .405 | 2.740 | 1 | .098 | 1.955 | .884 | 4.324 |
| 100 - 150 K v. < 50 K | .571 | .462 | 1.529 | 1 | .216 | 1.770 | .716 | 4.378 |
| > 150 K v. < 50 K | 1.320 | .478 | 7.614 | 1 | **.006** | 3.743 | 1.466 | 9.558 |
| Prefer not to say v. < 50 K | -.440 | .748 | .347 | 1 | .556 | .644 | .149 | 2.787 |
| Diagnosis |  |  | 15.863 | 2 | **<.001** |  |  |  |
| Autism v. Autism + ADHD | -.045 | .347 | .017 | 1 | .896 | .956 | .484 | 1.886 |
| ADHD v. Autism + ADHD | -1.329 | .382 | 12.113 | 1 | **<.001** | .265 | .125 | .560 |

Autism Domain = χ^2^(11) = 37.171, *p* <.001; Nagelkerke R^2^ = .183

Note: v. = versus; NZ = New Zealand; K = Thousand. Significant values (p ≤ 0.05) are in **bold.**

Supplementary Table 2.

*Logistic regression of ADHD across demographic and diagnostic categories*

|  | **ADHD Characteristics** | | | | | | | |
| --- | --- | --- | --- | --- | --- | --- | --- | --- |
| Predictors | β | S.E | Wald | df | *p* | Exp (β) | *95% C.I for Exp* (β) | |
|  |  |  |  |  |  |  | *Lower* | Upper |
| Age | -.008 | .004 | 3.679 | 1 | .055 | .992 | .984 | 1.000 |
| Gender |  |  | 5.129 | 2 | .077 |  |  |  |
| Male v. female | .255 | .390 | .427 | 1 | .513 | 1.290 | .601 | 2.772 |
| Gender diverse v. female | 2.466 | 1.089 | 5.129 | 1 | **.024** | 11.774 | 1.394 | 99.483 |
| Ethnicity |  |  | 2.384 | 2 | .304 |  |  |  |
| Māori v. NZ European | -.625 | .419 | 2.227 | 1 | .136 | .535 | .236 | 1.216 |
| Other v. NZ European | .129 | .599 | .046 | 1 | .830 | 1.138 | .351 | 3.683 |
| Level of Income |  |  | 3.898 | 4 | .420 |  |  |  |
| 50 - 100 K v. < 50 K | -.170 | .456 | .139 | 1 | .709 | .844 | .345 | 2.062 |
| 100 - 150 K v. < 50 K | .491 | .499 | .968 | 1 | .325 | 1.634 | .614 | 4.344 |
| > 150 K v. < 50 K | .536 | .522 | 1.056 | 1 | .304 | 1.709 | .615 | 4.751 |
| Prefer not to say v. < 50 K | -.161 | .730 | .049 | 1 | .825 | .851 | .204 | 3.557 |
| Diagnosis |  |  | 44.773 | 2 | **<.001** |  |  |  |
| Autism v. Autism + ADHD | -1.475 | .466 | 10.005 | 1 | **.002** | .229 | .092 | .571 |
| ADHD v. Autism + ADHD | 1.415 | .370 | 14.631 | 1 | **<.001** | 4.115 | 1.993 | 8.495 |

ADHD Domain = χ^2^(19) = 69.589, *p* <.001; Nagelkerke R^2^ = .332

Note: v. = versus; NZ = New Zealand; K = Thousand. Significant values (p ≤ 0.05) are in **bold.**

Supplementary Table 3.

*Logistic regression of Externalising domain across demographic and diagnostic categories*

|  | **Externalising Characteristics** | | | | | | | |
| --- | --- | --- | --- | --- | --- | --- | --- | --- |
| Predictors | β | S.E | Wald | df | *p* | Exp (β) | *95% C.I for Exp* (β) | |
|  |  |  |  |  |  |  | *Lower* | Upper |
| Age | -.002 | .003 | .291 | 1 | .590 | .998 | .992 | 1.005 |
| Gender |  |  |  |  |  |  |  |  |
| Male v. female | .633 | .351 | 3.258 | 1 | .071 | 1.883 | .947 | 3.745 |
| Gender diverse v. female |  |  |  |  |  |  |  |  |
| Ethnicity |  |  | 1.864 | 2 | .394 |  |  |  |
| Māori v. NZ European | -.249 | .347 | .514 | 1 | .473 | .780 | .395 | 1.540 |
| Other v. NZ European | .525 | .502 | 1.093 | 1 | .296 | 1.690 | .632 | 4.519 |
| Level of Income |  |  | .564 | 4 | .967 |  |  |  |
| 50 - 100 K v. < 50 K | -.027 | .386 | .005 | 1 | .944 | .973 | .457 | 2.075 |
| 100 - 150 K v. < 50 K | -.242 | .450 | .289 | 1 | .591 | .785 | .325 | 1.896 |
| > 150 K v. < 50 K | -.230 | .461 | .249 | 1 | .618 | .794 | .322 | 1.961 |
| Prefer not to say v. < 50 K | -.048 | .629 | .006 | 1 | .939 | .953 | .278 | 3.267 |
| Diagnosis |  |  | 6.096 | 2 | **.047** |  |  |  |
| Autism v. Autism + ADHD | -.805 | .357 | 5.066 | 1 | **.024** | .447 | .222 | .901 |
| ADHD v. Autism + ADHD | -.119 | .343 | .120 | 1 | .729 | .888 | .453 | 1.739 |

Externalising Domain = χ^2^(10) = 12.609, *p* = .246; Nagelkerke R^2^ = .067

Note: v. = versus; NZ = New Zealand; K = Thousand. Significant values (p ≤ 0.05) are in **bold.**

Supplementary Table 4.

*Logistic regression of Internalising domain across demographic and diagnostic categories*

|  | **Internalising Characteristics** | | | | | | | |
| --- | --- | --- | --- | --- | --- | --- | --- | --- |
| Predictors | β | S.E | Wald | df | *p* | Exp (β) | *95% C.I for Exp* (β) | |
|  |  |  |  |  |  |  | *Lower* | Upper |
| Age | .008 | .004 | 4.000 | 1 | **.045** | 1.008 | 1.000 | 1.015 |
| Gender |  |  | 1.428 | 2 | .490 |  |  |  |
| Male v. female | -.444 | .380 | 1.369 | 1 | .242 | .641 | .305 | 1.350 |
| Gender diverse v. female | -.103 | 1.023 | .010 | 1 | .920 | .902 | .122 | 6.697 |
| Ethnicity |  |  | 3.888 | 2 | .143 |  |  |  |
| Māori v. NZ European | .363 | .377 | .925 | 1 | .336 | 1.437 | .686 | 3.009 |
| Other v. NZ European | -1.749 | 1.067 | 2.687 | 1 | .101 | .174 | .021 | 1.408 |
| Level of Income |  |  | 2.611 | 4 | .625 |  |  |  |
| 50 - 100 K v. < 50 K | -.449 | .447 | 1.007 | 1 | .316 | .638 | .266 | 1.534 |
| 100 - 150 K v. < 50 K | .017 | .502 | .001 | 1 | .974 | 1.017 | .380 | 2.718 |
| > 150 K v. < 50 K | .192 | .514 | .140 | 1 | .708 | 1.212 | .443 | 3.318 |
| Prefer not to say v. < 50 K | -.516 | .802 | .414 | 1 | .520 | .597 | .124 | 2.874 |
| Diagnosis |  |  | 17.470 | 2 | **<.001** |  |  |  |
| Autism v. Autism + ADHD | 1.005 | .388 | 6.697 | 1 | **.010** | 2.733 | 1.276 | 5.851 |
| ADHD v. Autism + ADHD | -.756 | .486 | 2.420 | 1 | .120 | .470 | .181 | 1.217 |

Internalising Domain = χ^2^(11) = 31.667, *p* <.001; Nagelkerke R^2^ = .177

Note: v. = versus; NZ = New Zealand; K = Thousand. Significant values (p ≤ 0.05) are in **bold.**

Supplementary Table 5.

*Logistic regression of Emotional Dysregulation domain across demographic and diagnostic categories*

|  | **Emotional Dysregulation Characteristics** | | | | | | | |
| --- | --- | --- | --- | --- | --- | --- | --- | --- |
| Predictors | β | S.E | Wald | df | *p* | Exp (β) | *95% C.I for Exp* (β) | |
|  |  |  |  |  |  |  | *Lower* | Upper |
| Age | -.001 | .003 | .046 | 1 | .831 | .999 | .993 | 1.006 |
| Gender |  |  | 2.040 | 2 | .361 |  |  |  |
| Male v. female | -.371 | .322 | 1.330 | 1 | .249 | .690 | .367 | 1.296 |
| Gender diverse v. female | -1.248 | 1.166 | 1.146 | 1 | .284 | .287 | .029 | 2.822 |
| Ethnicity |  |  | 1.725 | 2 | .422 |  |  |  |
| Māori v. NZ European | .271 | .330 | .673 | 1 | .412 | 1.311 | .687 | 2.503 |
| Other v. NZ European | .573 | .505 | 1.286 | 1 | .257 | 1.774 | .659 | 4.774 |
| Level of Income |  |  | 6.829 | 4 | .145 |  |  |  |
| 50 - 100 K v. < 50 K | .103 | .370 | .078 | 1 | .780 | 1.109 | .537 | 2.291 |
| 100 - 150 K v. < 50 K | -.449 | .443 | 1.027 | 1 | .311 | .638 | .268 | 1.521 |
| > 150 K v. < 50 K | .171 | .440 | .151 | 1 | .698 | 1.186 | .501 | 2.810 |
| Prefer not to say v. < 50 K | -1.614 | .821 | 3.862 | 1 | **.049** | .199 | .040 | .996 |
| Diagnosis |  |  | .426 | 2 | .808 |  |  |  |
| Autism v. Autism + ADHD | -.191 | .344 | .307 | 1 | .580 | .827 | .421 | 1.622 |
| ADHD v. Autism + ADHD | -.009 | .349 | .001 | 1 | .979 | .991 | .500 | 1.962 |

Emotional Dysregulation Domain = χ^2^(11) = 11.981, *p* = .365; Nagelkerke R^2^ = .062

Note: v. = versus; NZ = New Zealand; K = Thousand. Significant values (p ≤ 0.05) are in **bold.**

Supplementary Table 6.

*Logistic regression of Sensory Issues domain across demographic and diagnostic categories*

|  | **Sensory Issues** | | | | | | | |
| --- | --- | --- | --- | --- | --- | --- | --- | --- |
| Predictors | β | S.E | Wald | df | *p* | Exp (β) | *95% C.I for Exp* (β) | |
|  |  |  |  |  |  |  | *Lower* | Upper |
| Age | -.001 | .004 | .018 | 1 | .893 | .999 | .992 | 1.007 |
| Gender |  |  | .010 | 2 | .995 |  |  |  |
| Male v. female | .033 | .391 | .007 | 1 | .933 | 1.033 | .480 | 2.224 |
| Gender diverse v. female | -.036 | 1.194 | .001 | 1 | .976 | .964 | .093 | 10.005 |
| Ethnicity |  |  | .534 | 2 | .766 |  |  |  |
| Māori v. NZ European | -.054 | .384 | .020 | 1 | .888 | .947 | .446 | 2.010 |
| Other v. NZ European | .395 | .574 | .472 | 1 | .492 | 1.484 | .481 | 4.574 |
| Level of Income |  |  | 3.142 | 4 | .534 |  |  |  |
| 50 - 100 K v. < 50 K | .105 | .421 | .062 | 1 | .803 | 1.110 | .487 | 2.532 |
| 100 - 150 K v. < 50 K | -.598 | .538 | 1.236 | 1 | .266 | .550 | .192 | 1.578 |
| > 150 K v. < 50 K | -.316 | .530 | .356 | 1 | .551 | .729 | .258 | 2.060 |
| Prefer not to say v. < 50 K | -.713 | .842 | .718 | 1 | .397 | .490 | .094 | 2.551 |
| Diagnosis |  |  | 4.533 | 2 | .104 |  |  |  |
| Autism v. Autism + ADHD | .045 | .383 | .014 | 1 | .906 | 1.046 | .494 | 2.217 |
| ADHD v. Autism + ADHD | -.780 | .442 | 3.117 | 1 | .077 | .458 | .193 | 1.090 |

Sensory Issues Domain = χ^2^(11) = 9.112, *p* = 612; Nagelkerke R^2^ = .054

Note: v. = versus; NZ = New Zealand; K = Thousand. Significant values (p ≤ 0.05) are in **bold.**

Supplementary Table 7

*Logistic regression of Communication domain across diagnostic categories only*

|  | **Communication Issues n = 16** | | | | | | | |
| --- | --- | --- | --- | --- | --- | --- | --- | --- |
| Predictors | β | S.E | Wald | df | *p* | Exp (β) | *95% C.I for Exp* (β) | |
|  |  |  |  |  |  |  | *Lower* | Upper |
| Diagnosis |  |  | 11.259 | 2 | **.004** |  |  |  |
| Autism v. Autism + ADHD | 2.512 | 1.047 | 5.754 | 1 | **.016** | 12.333 | 1.583 | 96.061 |
| ADHD v. Autism + ADHD | -.127 | 1.423 | .008 | 1 | .929 | .881 | .054 | 14.334 |

Communication Domain = χ^2^(2) = 18.081, *p* <.001; Nagelkerke R^2^ = .182

Note: v. = versus; Significant values (p ≤ 0.05) are in **bold.**

Supplementary Table 8

*Logistic regression of Sleep Issues domain selection (>10 cases) across diagnostic categories only*

|  | **Sleep Issues n = 23** | | | | | | | |
| --- | --- | --- | --- | --- | --- | --- | --- | --- |
| Predictors | β | S.E | Wald | df | *p* | Exp (β) | *95% C.I for Exp* (β) | |
|  |  |  |  |  |  |  | *Lower* | Upper |
| Diagnosis |  |  | .073 | 2 | .964 |  |  |  |
| Autism v. Autism + ADHD | -.018 | .529 | .001 | 1 | .973 | .982 | .348 | 2.771 |
| ADHD v. Autism + ADHD | -.137 | .560 | .060 | 1 | .806 | .872 | .291 | 2.611 |

Sleep Issues Domain = χ^2^(2) = .074, *p* =.964; Nagelkerke R^2^ = .001

Note: v. = versus; Significant values (p ≤ 0.05) are in **bold.**

Supplementary Table 9

*Logistic regression of Elopement domain selection (>10 cases) across diagnostic categories only*

|  | **Elopement n = 19** | | | | | | | |
| --- | --- | --- | --- | --- | --- | --- | --- | --- |
| Predictors | β | S.E | Wald | df | *p* | Exp (β) | *95% C.I for Exp* (β) | |
|  |  |  |  |  |  |  | *Lower* | Upper |
| Diagnosis |  |  | 3.301 | 2 | .192 |  |  |  |
| Autism v. Autism + ADHD | .808 | .605 | 1.783 | 1 | .182 | 2.244 | .685 | 7.351 |
| ADHD v. Autism + ADHD | -.132 | .726 | .033 | 1 | .856 | .877 | .211 | 3.634 |

Elopement Domain = χ^2^(2) = 3.365, *p* = .186; Nagelkerke R^2^ = .032

Note: v. = versus; Significant values (p ≤ 0.05) are in **bold.**
